# The effects of aqueous extract from watermelon (*Citrullus lanatus*) peel on the growth and physiological characteristics of *Dolichospermum flos-aquae*

**DOI:** 10.1038/s41598-022-12124-5

**Published:** 2022-05-16

**Authors:** Jin Yan, Peiyao Xu, Fengrui Zhang, Xinyue Huang, Yanmin Cao, Shenghua Zhang

**Affiliations:** 1 Key Laboratory of Resources Conversion and Pollution Control of the State Ethnic Affairs Commission, College of Resources and Environmental Science, South-Central Minzu University, Wuhan, 430074 People’s Republic of China; 2 Department of Civil and Environmental Engineering, Virginia Tech, Blacksburg, VA 24061 USA

**Keywords:** Environmental microbiology, Freshwater ecology, Environmental sciences

## Abstract

Nowadays, the increasing *Dolichospermum* (*Anabaena*) blooms pose a major threat to the aquatic environment and public health worldwide. The use of naturally derived chemicals from plants to control cyanobacteria blooms has recently received a tremendous amount of attention. This study investigates the possibility of transforming watermelon peel (WMP) into a biological resource to allelopathically inhibit *Dolichospermum flos-aquae* blooms. The results demonstrated that the growth of *D. flos-aquae* was efficiently restricted by the aqueous extract of watermelon peel (WMPAE) in a concentration-dependent manner. Cell viability decreased quickly, intracellular structural damage occurred, chlorophyll *a* in algal cells degraded, and photosynthesis was clearly inhibited. At the same time, the levels of reactive oxygen species in viable cells increased significantly, as did malondialdehyde levels, indicating that WMPAE elucidated strong oxidative stress and corresponding damage to *D. flos-aquae*. Capsular polysaccharide (CPS) levels increased in all treatment groups, which represents an adaptive response indicative of the development of resistance to WMPAE stress and oxidative damage. Despite this, WMPAE had clear inhibitory effects on *D. flos-aquae*. These findings provide fundamental information on an allelopathic system that could be a novel and attractive approach for suppressing *D. flos-aquae* blooms in small aquatic environments, especially aquaculture ponds.

## Introduction

Toxic cyanobacterial blooms have adverse influences on aquatic ecosystems and water quality^1,2^. Specifically, the toxins released by cyanobacteria can harm other aquatic creatures and endanger human health^3–5^. *Dolichospermum* (*Anabaena*), *Microcystis,* and *Aphanizomenon* are the most common species predominating cyanobacterial blooms^6^. *Dolichospermum flos-aquae* is one of the primary cyanobacterial species in Chaohu Lake in China^7^, with its blooms usually occurring during spring, autumn, and winter^8^. The *D. flos-aquae* cells can produce various toxins, such as microcystin, anatoxin-a (s), and anatoxin-a^9–12^. Recently, the frequency and persistence of these blooms have increased, posing a great threat to the water environmental quality and public health. Therefore, there is an exigent necessity to develop practical, economical, and environmentally friendly approaches for the control and elimination of cyanobacteria blooms, including those dominated by *D. flos-aquae*.

In recent years, methods to use plant’s naturally produced chemicals to control cyanobacteria blooms have been suggested due to an increasing in-depth cognition of the allelopathic effects of plants^13–16^. The advantage of using chemicals derived from plants is that they are easy to degrade (making them environmentally friendly) and are very effective at suppressing algal growth^13,17^. The elimination and control of cyanobacteria bloom by plant-derived algaecides has been explored rather extensively for freshwater environments. Notably, barley straw has been applied in field and laboratory trials to suppress *Microcystis* blooms^13,18^. Aqueous extracts from *Spartina alterniflora* has also been utilized to control *Microcystis aeruginosa* blooms^16^. Furthermore, eucalyptus extract, an algicide, has the potential to control *M. aeruginosa*^19^. However, in comparison with *Microcystis*, the inhibitory effects of plant-derived chemicals on *Dolichospermum* spp. have yet to be extensively researched^20^.

Watermelon (*Citrullus lanatus*) is a sweet, popular fruit, and its peel is an agricultural waste product. Our previous study indicated that watermelon peel (WMP) can restrain the expansion of *Aphanizomenon flos-aquae*^21^. However, we did not investigate the potential of the aqueous extract of watermelon peel (WMPAE) for *D. flos-aquae* control. Hence, we have examined the inhibitory effects and potential mechanisms of WMPAE on *D. flos-aquae* by monitoring changes in cell viability, intracellular structures, pigments, photosynthetic activities, malondialdehyde (MDA), reactive oxygen species (ROS), and capsular polysaccharides (CPS).

## Materials and methods

### WMPAE preparation and *D. flos-aquae* cultivation

The most popular and highly productive watermelon named Ningxia stone watermelon, which is available in months between July to September, were purchased from a fruit market to obtain WMPs. After removing the thin rind layer (approximately 0.1 cm), WMPs were sliced into approximately 3–4 cm pieces, and immersed thoroughly with pure water. The washed WMPs were dried in a blast oven at 45 °C, and then frozen at − 20 °C. Following this, 12 g of dried WMP was soaked in 1 L axenic water to begin the extraction process, which lasted for 3 d at 25 °C. Finally, 0.012 g mL^−1^ WMPAE was obtained after nutrient addition and the removal of residues and microorganisms by filtration through a PES membrane (0.22 μm, Millipore Millex-GP).

The target cyanobacterial species *D. flos-aquae* (FACHB 245) was purchased from the Freshwater Algae Culture Collection of the Institute of Hydrobiology, Chinese Academy of Sciences. *D. flos-aquae* cells were cultivated in sterile BG-11 solution in a light incubator (25 ± 1 °C, 64 μmol photons m^−2 ^s^−1^) under a well-controlled 14 h:10 h light/dark cycle. All culture operations were performed in a clean environment, using Erlenmeyer flasks and all materials that could be were sterilized in an autoclave at 121 °C for 30 min. Algal cells in logarithmic growth phase were inoculated at the start of each experiment.

### Bioassays of the effects of WMPAE on *D. flos-aquae*

*D. flos-aquae* solution (80 mL; cell density of 7 × 10^7^ cells mL^−1^ prepared from cells in exponential growth) was transferred into axenic 250-mL Erlenmeyer flasks. Then, 24, 48, or 72 mL of WMPAE (0.012 g mL^−1^) was added with sterile BG-11 medium to reach a total volume of 160 mL. The corresponding concentrations of WMPAE were designated the 1.8 g L^−1^, 3.6 g L^−1^, and 5.4 g L^−1^ treatment groups. The initial algal density for each experiment was approximately 3.5 × 10^7^ cells mL^−1^. Cultures without WMPAE served as a control group (0 g L^−1^ group). Each group had three replicates. All flasks were maintained in a light incubator (64 μmol photons m^−2 ^s^−1^) at 25 ± 1 °C under the light/dark cycle of 14 h:10 h. All culture flasks were shaken 3 times daily, and their position was varied randomly to decrease any potential light influence on the results.

### Determination of algal cell density and pigments

A microscope (Olympus CX31, Japan) and hemocytometer were used to count the cell number every 24 h, each vegetative cell was defined as a one-unit cell. Pigments were also measured every 24 h over the experimental period. Algal cells in 5 mL culture solution were condensed through centrifugation (8000× *g* for 10 min). The collected cells were resuspended in 5 mL cold 80% acetone, and then placed in the dark at 4 °C for 24 h to extract chlorophyll a (Chl a) and carotenoid. Finally, the contents of Chl a and carotenoid were measured and calculated using the formula described by Lichtenthaler and Buschmann^22^.

In vivo single-cell Raman spectra were also used to describe the variation in carotenoid content. Culture solutions (1 mL) were centrifuged for 5 min at 1500× *g*, and the cell pellets were resuspended in 0.3 mL PBS solution after washing with 0.01 M PBS solution (pH 7.2–7.4) twice. Following this, the micro-Raman spectra of *D. flos-aquae* cells were measured on a DXR Raman Microscope (ThermoFisher, America) excited by a 532 nm laser with 7 mW at 25 ± 1 °C. The laser was focused on *D. flos-aquae* cells using a × 10 objective. All spectra were collected twice with an acquisition time of 5 s, and wavenumber range of 600–1800 cm^−1^.

### Transmission electron microscopy (TEM) assays

Culture solution (35 mL) was centrifuged for 15 min at 1500× g to collect *D. flos-aquae* cell samples. These were fixed and dehydrated, and ultrathin sections were prepared using the procedures depicted by Ozaki et al.^23^. Examination of the stained samples was performed on a transmission electron microscope (Hitachi 7700, Japan). Representative images of *D. flos-aquae* cells treated with 5.4 g L^−1^ WMPAE at 24 h and 48 h were retained for presentation.

### Observations and measurements of cell viability

A quantitative measurement of cell viability was performed on a 96-well microplate using CCK-8 assay (Cell Counting Kit-8, Beyotime Biotechnology, China). Firstly, the culture solutions were condensed through centrifugation (1500× *g* for 15 min). The collected cell pellets were further washed using 0.01 M PBS solution (pH 7.2–7.4), and then resuspended in PBS solution to obtain an algal solution with a density of 100,000 cells mL^−1^. Following this, 10 μL of CCK-8 solution and 100 μL algal solution (containing 10,000 cells) were combined in a microplate well. After shaking for 5 min in the dark on a micro-vibrator, the 96-well microplate was put into an incubator for 2 h under darkness at 35 °C. Finally, a microplate spectrophotometer (Spark 10 M, Tecan, Switzerland) was used to measure the absorbance of each microplate well at 450 nm. The cell viability of *D. flos-aquae* in the treatment groups was quantified according to the following equation: ${\text{Cell}}\;{\text{viability}}\,(\%) = { }\frac{A_{\text{Treatment}} - A_{\text{Blank}}}{A_{\text{Control}} - A_{\text{Blank}}} \times 100$, where A_Blank_ represents wells containing PBS only, and A_Treatment_ and A_Control_ are the absorbances of the wells with treated cells and control cells, respectively.

The fluorescent images of *D*. *flos-aquae* cells in the control group and treatment groups were investigated for cell viability. *D*. *flos-aquae* cells were stained with fluorescein diacetate (FDA, Sigma-Aldrich) following to the method of Zhang et al.^21^, and fluorescent images of *D*. *flos-aquae* were acquired on a fluorescence microscope using a 40 × objective with 494 nm excitation and 521 nm emission (Zeiss Axio Observer 7, Germany).

### Determination of MDA, ROS, and CPS levels

The sample preparation process for MDA determination was as follows: algal culture was centrifugated for 20 min at 4 °C at 10,800× *g*, the harvested cell pellets were then diluted using 2 mL PBS (0.005 M, pH 7.2) and disrupted with an ultrasonic cell pulverizer at 300 W, in an ice-bath with 5 s bursts and 3 s of rest time for 5 min (Scientz Biotechnology Co., Ltd., China). The broken homogenate was centrifugated at 14,800× *g* for 20 min at 4 °C to achieve a cell-free supernatant, which was used to determine MDA contents following the manufacturer’s instructions. Commercial MDA assay kits (Nanjing Jiancheng Bioengineering Institute, China) were used to assess MDA contents.

Commercialized ROS assay kits, produced by Nanjing Jiancheng Bioengineering Institute, were purchased to measure ROS levels in *D*. *flos-aquae* cells. The culture solution was centrifuged for 15 min at 1500× *g* and washed twice with PBS (0.01 M, pH 7.2–7.4). Following which the pellet was resuspended in PBS to achieve an algal density of 100,000 cells mL^−1^, and 100 μL of the suspended sample was transferred to a 96-well microplate for incubation and determination of the ROS level, following the manufacturer’s instructions. The fluorescence intensity was determined using a Spark 10 M multimode microplate reader (Tecan, Switzerland) with excitation at 485 nm and emission at 530 nm. ROS levels in the treatment groups versus the control group (relative ROS level, %) were calculated as follows: ${\text{Relative }}\;{\text{ROS}}\;{\text{level }}\left( {{\% }} \right) = \frac{{{\text{Fluorescence}}\left[ {{\text{Treatment}}} \right]}}{{{\text{Fluorescence}}\left[ {{\text{Control}}} \right]}} \times 100$.

Capsular polysaccharide (CPS) is the major chemical component of algal mucilaginous sheaths. CPS extraction from *D*. *flos-aquae* cells was performed according to the method of Staats and coworkers^24^. The extracted CPS was measured using the phenol–sulfuric acid method^25^.

### Determination of chlorophyll fluorescence parameters

The chlorophyll fluorescence parameters of *D. flos-aquae* (3 mL) were determined on a pulse amplitude modulated fluorometer (FMS-2, Hansatech Instruments, Norfolk, UK) using the method described by Zhang et al.^21^. The maximum quantum yield (${\text{Fv}}/{\text{Fm}}$) of photosystem II (PSII), the effective PSII quantum yield (${\text{F}}_{{\text{v}}}^{^{\prime}} /{\text{F}}_{{\text{m}}}^{^{\prime}}$), and non-photochemical quenching (NPQ) were calculated basing on the equations from Roháček and Barták^26^.

### Data analysis

All control and treatment groups were performed in triplicate, data are presented as the mean ± standard deviation (SD). Independent-sample *t*-tests and Shapiro–Wilk tests were implemented in SPSS software (13.0, USA) to test the normality of the data and to detect differences between the treatment groups and control group, respectively. A result with *p* ≤ 0.05 was considered statistically significant. The units examined for cell viability, CPS, MDA, ROS, and pigment levels were defined using the cell-counting method. The growth inhibition efficiency of the WMPAE on *D. flos-aquae* was estimated by:

$${\text{Inhibition}}\;{\text{efficiency }}\left( {{\% }} \right) = { }\frac{{({\text{Mean }}\;{\text{Cell}}\;{\text{density}}_{{{\text{control}}}} - {\text{Cell}}\;{\text{density}}_{{{\text{treatment}}}} )}}{{{\text{Mean}}\;{\text{Cell}}\;{\text{density}}_{{{\text{control}}}} }}{ } \times 100.$$

## Results and discussion

### Antialgal activity of WMPAE

**Figure 1:**
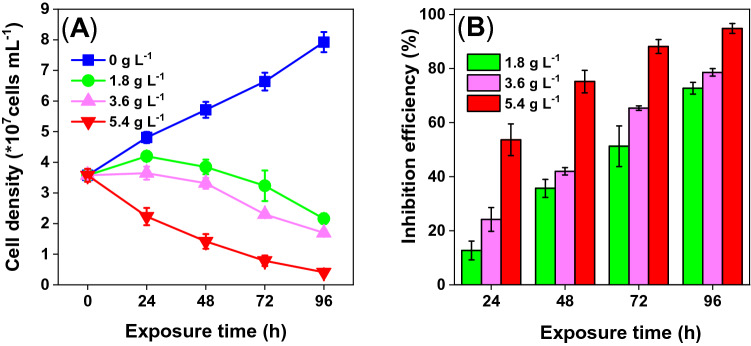
Growth curves (**A**) and algal growth inhibition efficiency (**B**) of the *Dolichospermum flos-aquae* after treatment with different aqueous extract of watermelon peel (WMPAE) concentrations. The data in the figures are shown as mean ± standard deviation (n = 3).

The growth curves of *D. flos-aquae* and the algal inhibition efficiency of WMPAE are presented in Fig. 1. The *D. flos-aquae* in the 0 g L^−1^ group (control) grew quickly during the cultural period, with cell density increasing from 3.5 × 10^7^ at 0 h to 7.93 × 10^7^ cells mL^−1^ at 96 h (Fig. 1A). The growth of *D. flos-aquae* was inhibited in all three WMPAE treatment groups, with both time- and concentration-dependent trends observed during the 96-h exposure period. Growth inhibition was obvious in all three treatment groups from 24 h onward, with inhibition efficiencies of 13%, 24%, and 54% for the 1.8 g L^−1^, 3.6 g L^−1^, and 5.4 g L^−1^WMPAE treatments, respectively (Fig. 1B). Pronounced algicidal effects were noted at 96 h, with inhibitions of 73%, 79%, and 95% for cells exposed to 1.8 g L^−1^, 3.6 g L^−1^, and 5.4 g L^−1^ WMPAE, respectively (*p* ≤ 0.05, Fig. 1B). It has been reported that extracts from *Hydrilla verticillate* can suppress the growth of *D. flos-aquae*^27^, as can compounds from the *Bacillus cereus* strain^28^. Moreover, Kaminski et al.^29^ suggested that *Lemna trisulca* could naturally eliminate *D. flos-aquae*. Similarly, the present results showed that the growth of *D. flos-aquae* could be inhibited efficiently by WMPAE. WMP is a common agricultural waste product. However, there were different watermelon varieties. So, in the pre-experiments, all the WMPs of various types available in the fruit market during winter to summer were tested and the results showed reproducible the inhibition effects (data not shown). Furthermore, our previous investigations revealed that *A. flos-aquae* could be suppressed by WMPAE^21^. The ability to also inhibit the growth of *D. flos-aquae* shows it has promise as a *D. flos-aquae* bloom control method.

### Cell viability of D. flos-aquae

**Figure 2:**
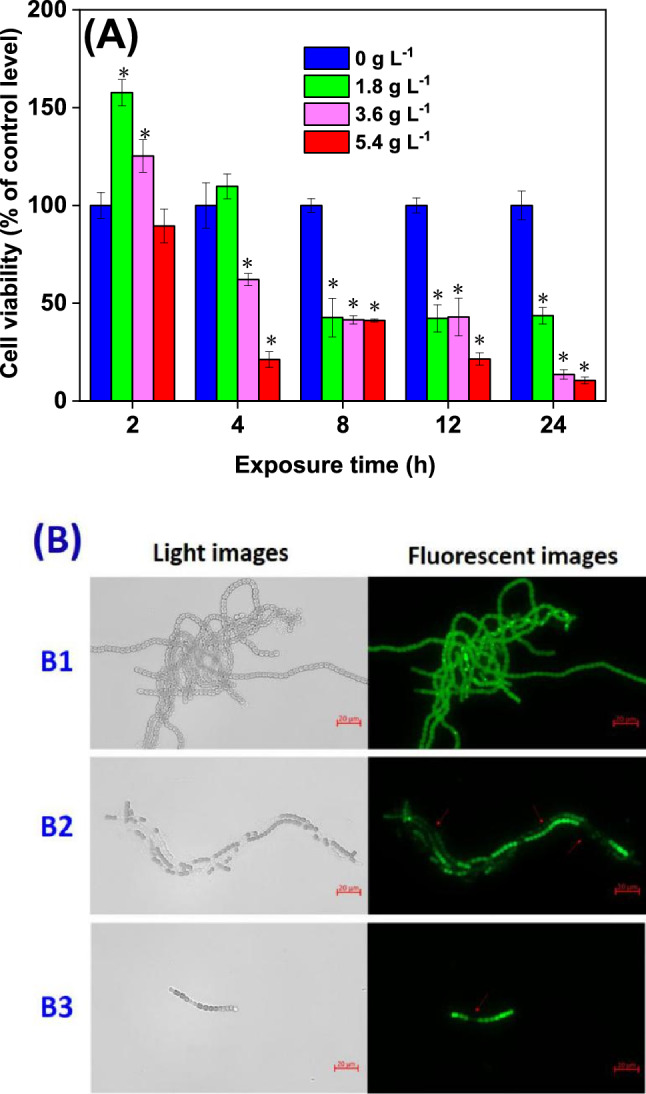
Cell viability of *Dolichospermum flos-aquae* treated with aqueous extract of watermelon peel (WMPAE): (**A**) Quantitative measurement results (Data are shown as mean ± standard deviation for n = 3, * indicates *p* < 0.05); (**B**) Light images and fluorescent images of algal cells in 0 g L^−1^ and 5.4 g L^−1^ WMPAE group: B1, cells in 0 g L^−1^ group; B2, cells in 5.4 g L^−1^ group for 24 h; B3, cells in 5.4 g L^−1^ group for 48 h.

The cell viability in *D. flos-aquae* treated with WMPAE was determined quantitatively by a CCK-8 assay (Fig. 2A) and observed qualitatively using fluorescence microscopy (Fig. 2B). In the 1.8 g L^−1^ and 3.6 g L^−1^ WMPAE treatment groups, cell viability showed an obvious increase over the first 2 h of exposure before subsequently decreasing. The viability in treated *D. flos-aquae* cells decreased to 43.7%, 13.6%, and 10.5% of the control level after 24 h of exposure to 1.8 g L^−1^, 3.6 g L^−1^, and 5.4 g L^−1^ WMPAE, respectively. Fluorescence images were obtained after staining algal cells with FDA. In the control group (0 g L^−1^ WMPAE), the filaments were very long and emitted strong green fluorescence (Fig. 2B1), indicating healthy algal cells. After exposure to 5.4 g L^−1^ WMPAE, some cells in the filaments had lost their viability (Fig. 2B2) and the filaments became shorter as exposure prolonged (Fig. 2B3). Decreased cell viability can serve as a basic indicator of dying algal cells^30^. Some chemicals, such as gallic acid and H_2_O_2_, can lead to the loss of cell viability in cyanobacteria^31,32^. Our results indicate that WMPAE can efficiently and promptly reduce *D. flos-aquae* cell viability.

### Intracellular structure of *D. flos-aquae*

**Figure 3:**
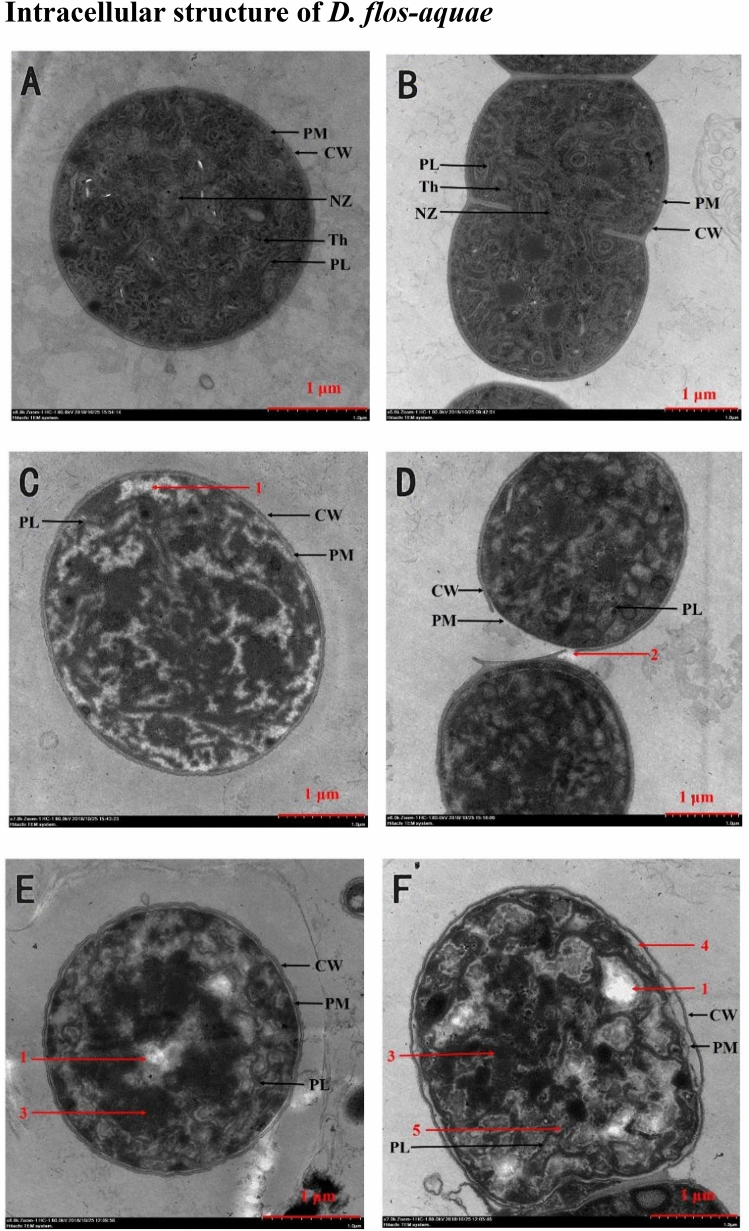
Representative TEM images of *Dolichospermum flos-aquae* cells treated with 0 g L^−1^ and 5.4 g L^−1^ aqueous extract of watermelon peel (WMPAE). (**A**) (**B**), normal cells in 0 g L^−1^ WMPAE group. (**C**,**D**), cells in 5.4 g L^−1^ WMPAE group after 24 h exposure. (**E**,**F**), cells in 5.4 g L^−1^WMPAE group after 48 h exposure. (Abbreviations and arrows number indicate: plasma membrane, (PM); cell wall, (CW); photosynthetic lamellae, (PL); nucleoid zone, (NZ); Thylakoid, (Th); 1, vacuolation; 2, disconnection between cells in filaments; 3, pyknosis formation; 4, the separation of cell wall and plasma membrane; 5, blurring of photosynthetic lamellae).

The intracellular structure of cyanobacteria could be destroyed by many allelochemicals^33,34^. Therefore, we examined the ultrastructure of *D. flos-aquae* cells in 0 g L^−1^ and 5.4 g L^−1^ WMPAE groups after 24 h and 48 h of exposure. Representative images taken by TEM are presented in Fig. 3. It is shown in Fig. 3A,B that the *D. flos-aquae* cells in the 0 g L^−1^ WMPAE group retained their structural integrity. The thylakoids (Th), a uniform cell wall (CW), plasma membrane (PM), photosynthetic lamellae (PL), and a normal nucleoid zone (NZ) were observed. However, the intracellular structures of *D. flos-aquae* cells treated with WMPAE were distinctly damaged (Fig. 3C–F). After 24 h of exposure, the intracellular structure became uneven, the NZ disappeared, and vacuolation was observed (see arrow 1 in Fig. 3C). We also observed clear disconnections between cells in the filaments (see arrow 2 in Fig. 3D), which contributed to the gradual shortening of the long *D. flos-aquae* filaments. Intracellular structure damage of *D. flos-aquae* was even prominent after 48 h of exposure, in which vacuolation (arrow 1) and pyknosis (arrow 3) were both observed (Fig. 3E,F). Moreover, the plasma membranes and cell walls were separated (arrow 4 in Fig. 3F), and the photosynthetic lamellae became blurred (arrow 5 in Fig. 3F). These phenomena demonstrate that the cell structures of *D. flos-aquae* were damaged by the WMPAE.

Through TEM analysis, it was demonstrated that *M. aeruginosa* cells in pyrogallic acid experience nucleoid disintegration, pyknosis, vacuolation, and photosynthetic lamellae rupture^33^. Moreover, Chen et al.^34^ reported that vitamin C could induce extensive damage of the cell walls, membranes, and thylakoid structure in *M. aeruginosa*. Additionally, in our previous study, WMPAE could induce intracellular structural damage in *A. flos-aquae*^21^. Similarly, *D. flos-aquae* cells also displayed structural damage after exposure to WMPAE. The intracellular structure became inhomogeneous firstly over time, along with the vacuolization and the disappearance of NZ. Pyknosis and the separation of the plasma membrane and cell wall occurred subsequently. In addition, the photosynthetic lamellae became blurred. On the one hand, these phenomena indicated that the cell structure of *D. flos-aquae* was damaged by WMPAE. On the other hand, the gradual changes of algal cells were considered as a process of *D. flos-aquae* being damaged by WMPAE, and therefore, the specific broken inclusion structures apparent in the TEM images of *D. flos-aquae* cells provide insight into the basic inhibitory mechanisms of WMPAE.

### ROS levels and lipid peroxidation of *D. flos-aquae*

**Figure 4:**
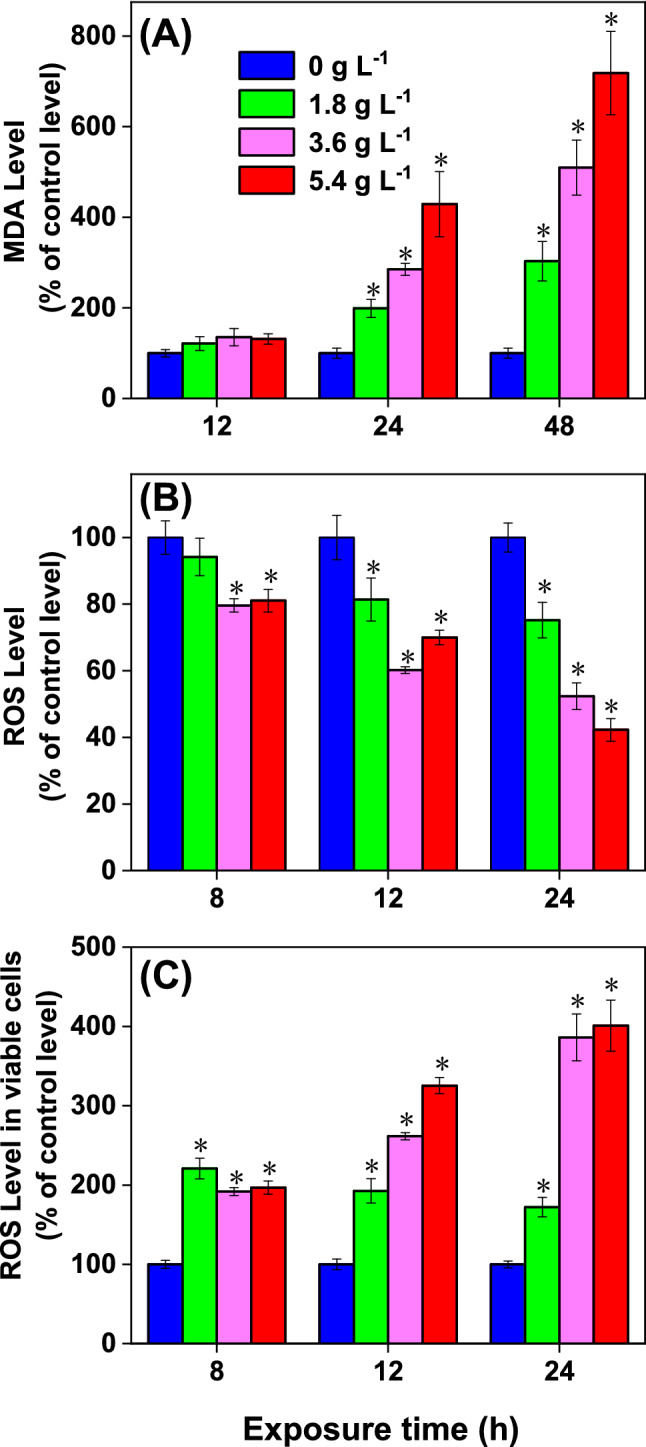
MDA level (**A**), ROS level (**B**), and ROS level in viable cells (**C**) of the *Dolichospermum flos-aquae* treated with aqueous extract of watermelon peel (WMPAE) at different concentrations. (Data are shown as mean ± standard deviation for n = 3, * indicates *p* < 0.05).

Oxidative stress can lead to cellular damage through undermining the basic functioning of some molecules and intracellular balance^35^. MDA could act as a good indicator of lipid peroxidation and oxidative stress in cyanobacteria^36,37^. In the present study, MDA and intracellular ROS levels were measured to evaluate the oxidative stress in *D. flos-aquae* under WMPAE (Fig. 4). Treatment with WMPAE significantly accelerated MDA accumulation in cyanobacteria cells in a dose-dependent and time-dependent manner (Fig. 4A). The MDA concentrations after 48 h of exposure to 1.8 g L^−1^, 3.6 g L^−1^, and 5.4 g L^−1^ WMPAE were 3.0 times, 5.1 times, and 7.2 times, respectively, than that of the control group. Abnormal lipid peroxidation in algal cells caused by bioactive compounds have been observed by many researchers^18,38^, including an MDA level increase in *M. aeruginosa* after luteolin exposure^38^. Furthermore, it was also found that the MDA levels of *A. flos-aquae* cells^39^ and *M. aeruginosa* cells^40^ increase greatly after treatment with *Sagittaria trifolia* extract. In this study, the significant increase in the MDA level of *D. flos-aquae* was also observed during exposure to WMPAE. This result clearly demonstrates heavy oxidative stress and severe damage to cell membranes by WMPAE^37^.

In contrast to the results for MDA, the ROS levels in all treatment groups decreased gradually during the first 24 h of exposure (Fig. 4B). The enhanced MDA level (Fig. 4A) of *D. flos-aquae* with increasing WMPAE was a distinct indication of intracellular oxidative stress, which is commonly caused by increased ROS production^41^. Considering the large decreases observed in the cell viability of *D. flos-aquae*, the lower ROS levels might be due to the dramatic loss of viable cells after treatment with WMPAE^42,43^. Therefore, the ROS levels in the viable cells treated with different concentrations of WMPAE were recalculated based on the cell viability (Fig. 4C). Compared to the viable cells in the control group, ROS levels in viable *D. flos-aquae* cells of the treatment groups increased significantly. This result was demonstrated time-dependent effects, increasing to 172%, 386%, and 401% of the control level after 24 h of exposure to 1.8 g L^−1^, 3.6 g L^−1^, and 5.4 g L^−1^ WMPAE, respectively. An enhanced ROS level can destroy normal metabolic function and lead to cell death^35,42^. Our quantitative results showed that in viable cells, both ROS and MDA levels markedly increased, indicating that the WMPAE can induce oxidative stress in *D. flos-aquae* cells. These findings are similar to the observations of Mecina and coworkers^18^, who reported that *Hordeum vulgare* extract could induce a rise in ROS levels in *M. aeruginosa*.

### Pigments of *D. flos-aquae*

**Figure 5:**
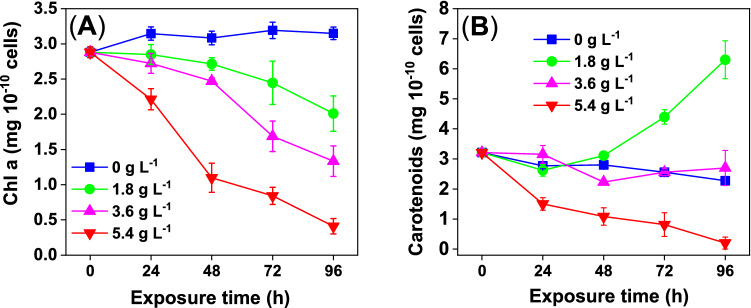
Chlorophyll a (Chl a) and carotenoid in *Dolichospermum flos-aquae* cells under the stress of aqueous extract of watermelon peel (WMPAE) (Data are shown as mean ± standard deviation for n = 3).

The WMPAE significantly affected the Chl a and carotenoid concentrations in *D. flos-aquae* cells (Fig. 5). Chl a is one of the major light-harvesting pigments in cyanobacterial cells^44^. Chl a reduction in cyanobacteria induced by allelochemicals or other environmental stressors have previously been reported^45,46^. We observed an evident Chl a reduction in treated *D. flos-aquae* in a dose-dependent and time-dependent manner (Fig. 5A). In the 0 g L^−1^ WPMAE group (control), the Chl a concentration was maintained steadily at 3.1 mg 10^–10^ cells during the 96-h exposure period. After 72 h of exposure, the Chl a concentrations of *D. flos-aquae* in the 1.8 g L^−1^, 3.6 g L^−1^, and 5.4 g L^−1^ WMPAE group reduced to 76%, 53%, and 26% of the control group level, respectively. The maximum decrease in Chl a (to 14% of the control level) was observed in the 5.4 g L^−1^ WMPAE treatment group after 96 h of exposure. These results imply that WMPAE affects the photosynthetic pigment of *D. flos-aquae* significantly, and that Chl a is susceptible to degradation under WMPAE-induced stress^47^.

**Figure 6:**
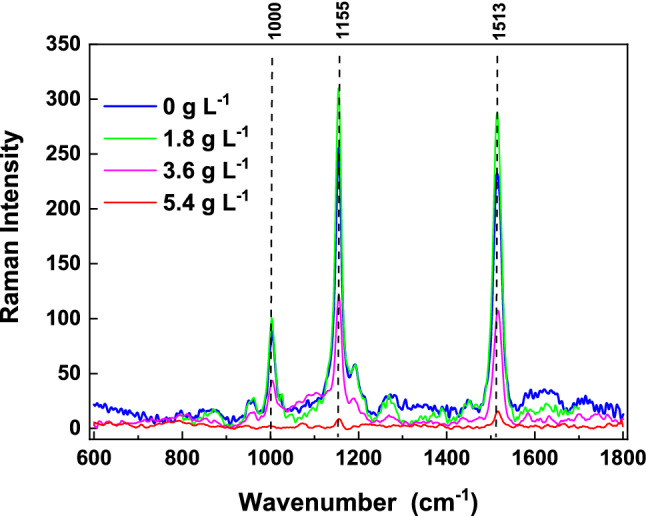
In vivo single-cell Raman spectra of *Dolichospermum flos-aquae* under aqueous extract of watermelon peel (WMPAE) stress.

Unlike Chl a, carotenoid concentrations in treated *D. flos-aquae* varied throughout the exposure period (Fig. 5B). In the 1.8 g L^−1^ group, the carotenoid concentration decreased slightly over 24 h; however, it exceeded that of the control group from 48 h onward, reaching a maximum value of 6.3 mg 10^–10^ cells at 96 h. In the 3.6 g L^−1^ group, the carotenoid concentration remained stable, similar to the control group. The carotenoid concentration in the 5.4 g L^−1^ group decreased significantly, reaching 0.20 mg 10^–10^ cells at 96 h, which represented 8.8% of the control group level. Raman spectra of *D. flos-aquae* cells produced by in vivo single-cell Raman spectroscopy confirmed the variations in carotenoids (Fig. 6). In the Raman spectra, 1513 cm^−1^ was assigned to conjugated C = C stretching vibrations, 1155 cm^−1^ was allocated to C–C vibrations coupled to C-CH_3_ stretches or C-H in-plane bending, and 1000 cm^−1^ was allotted to CH_3_ stretching modes, these data were considered characteristics of carotenoids^48,49^. The peak intensities were inhibited significantly in *D. flos-aquae* cells treated with 3.6 g L^−1^ and 5.4 g L^−1^ WMPAE, while those of *D. flos-aquae* cells treated with 1.8 g L^−1^ WMPAE were markedly enhanced.

As carotenoids have outstanding antioxidant activities, their increase is indicative of the cells’ remarkable capacity to resist oxidative stress and dissipate excited energy in non-photochemical ways^50,51^. In this study, the obvious carotenoid rises in *D. flos-aquae* cells treated with 1.8 g L^−1^ WMPAE hinted that WMPAE may bring oxidative stress and strengthen the pressure on energy dissipation in *D. flos-aquae*^52^. However, the carotenoid concentrations of *D. flos-aquae* treated by 45% WMPAE were obviously decreased, that may invalidate such a boycott mechanism at high exposure levels^52^.

### Photosynthetic activities of *D. flos-aquae*

**Figure 7:**
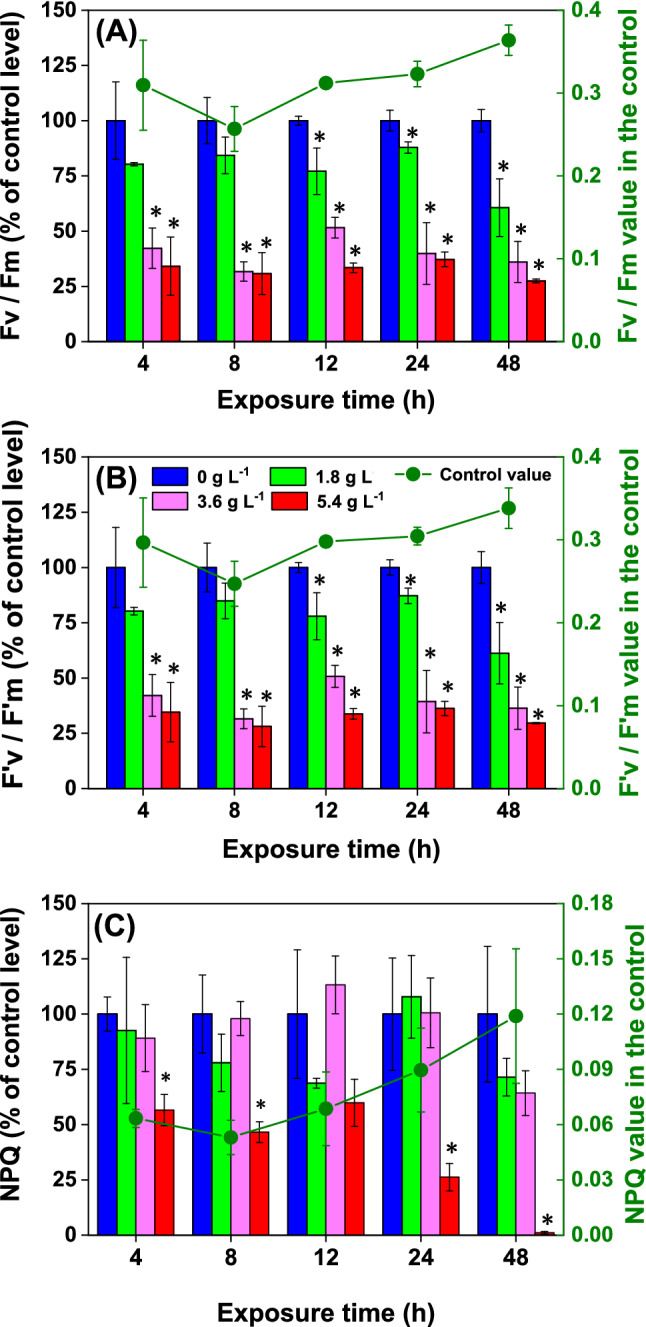
The ${\text{Fv}}/{\text{Fm}}$, ${\text{F}}_{{\text{v}}}^{\prime } /{\text{F}}_{{\text{m}}}^{\prime }$ and ${\text{NPQ}}$ of *Dolichospermum flos-aquae* treated with aqueous extract of watermelon peel (WMPAE) at different concentrations. (Data are shown as mean ± standard deviation for n = 3, * indicates *p* < 0.05).

Changes in algal photosynthesis under stress can be reflected in chlorophyll fluorescence parameters^53,54^. The parameters ${\text{Fv}}/{\text{Fm}}$, ${\text{F}}_{{\text{v}}}^{\prime } /{\text{F}}_{{\text{m}}}^{\prime }$, and NPQ of PSII are widely used as perceptive indicators of intracellular damage in cyanobacteria^55–57^. Figure 7 presented the changes in PSII ${\text{Fv}}/{\text{Fm}}$, ${\text{F}}_{{\text{v}}}^{\prime } /{\text{F}}_{{\text{m}}}^{\prime }$, and NPQ of *D*. *flos-aquae* induced by WMPAE. Significant inhibition of both ${\text{Fv}}/{\text{Fm}}$ and ${\text{F}}_{{\text{v}}}^{\prime } /{\text{F}}_{{\text{m}}}^{\prime }$ was observed in a dose-dependent and time-dependent manner. As shown in Fig. 7A,B, the ${\text{Fv}}/{\text{Fm}}$ and ${\text{F}}_{{\text{v}}}^{\prime } /{\text{F}}_{{\text{m}}}^{\prime }$ of *D*. *flos-aquae* declined promptly after 4 h of exposure to 3.6 g L^−1^ and 5.4 g L^−1^ WMPAE. After 48 h of exposure, the photosynthetic yield of *D*. *flos-aquae* in the 1.8 g L^−1^, 3.6 g L^−1^, and 5.4 g L^−1^ WMPAE treatment groups was significantly inhibited by 39%, 64%, 72% (for ${\text{Fv}}/{\text{Fm}}$) and 39%, 63%, 70% (for ${\text{F}}_{{\text{v}}}^{\prime } /{\text{F}}_{{\text{m}}}^{\prime }$), respectively. Significant decreases in photosynthetic yield of *D. flos-aquae* were observed after exposure to WMPAE, this result has also been reported in previous studies^21,58^. The photosynthetic yield of PSII in *M. aeruginosa* could be reduced significantly by metribuzin^56^. Dramatic decreases in the PSII photosynthetic yield of *Microcystis flos-aquae* were also observed under the stress of erythromycin^58^. Moreover, the ${\text{Fv}}/{\text{Fm}}$ and ${\text{F}}_{{\text{v}}}^{\prime } /{\text{F}}_{{\text{m}}}^{\prime }$ values in *M. aeruginosa* were decreased by ferulic acid^59^. Additionally, the filtrate from *Alexandrium minutum* could intensely inhibit the quantum yield of PSII in *Chaetoceros muelleri*^60^. ${\text{Fv}}/{\text{Fm}}$ and ${\text{F}}_{{\text{v}}}^{\prime } /{\text{F}}_{{\text{m}}}^{\prime }$ reflect the photosystem capacity of light conversion into chemical energy, and their reduction in *D. flos-aquae* cells treated by WMPAE indicates an inhibited conversion capacity^41,59^.

Non-photochemical quenching (NPQ) is a safely and non-radiatively deactivation process of excess excitation energy in PSII, which may aid in avoiding photo-damage in the photosynthetic system^44,54,61^. NPQ could be used as one of the most apposite signals of inhibitory effects due to its high susceptibility and response speed^57,62^. Compared with photosynthetic yield, the NPQ of *D. flos-aquae* changed in a slightly different manner after exposure to WMPAE. Specifically, a palpable reduction in the NPQ value of *D. flos-aquae* was only demonstrated in the 5.4 g L^−1^ WMPAE treatment group (Fig. 7C). Furthermore, the NPQ of *D. flos-aquae* in the 5.4 g L^−1^ WMPAE treatment group was almost entirely suppressed after 48 h of treatment. The dramatic reduction in the NPQ of *D. flos-aquae* under a high exposure level indicates the failure of this photoprotective mechanism^63,64^.

### CPS levels of *D. flos-aquae*

**Figure 8:**
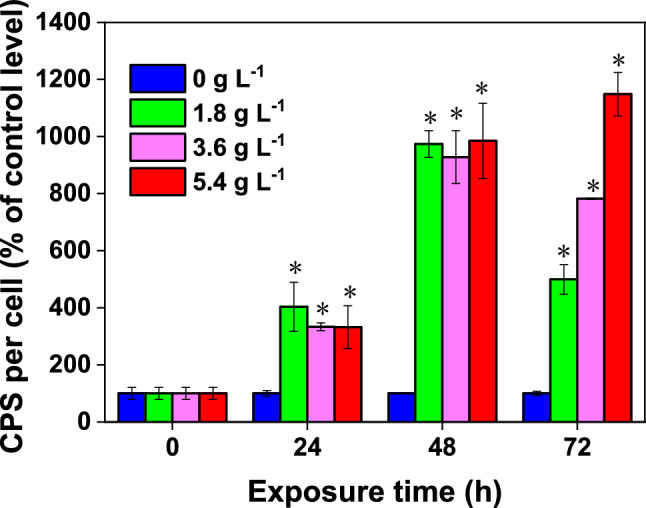
Capsular polysaccharide (CPS) levels in *Dolichospermum flos-aquae* treated with aqueous extract of watermelon peel (WMPAE) at different concentrations. (Data in the figure are shown as mean ± standard deviation for n = 3, *indicates *p* < 0.05).

Capsular polysaccharide (CPS) is capsular EPS which may help to build a protective layer (a mucilage sheath) that keeps algal cells in an isolated microenvironment to assist the cells resist poisonous substances or other environmental stressors^65^. Algae adapt to stress and defend themselves by increasing their EPS content^43^. It has been reported that EPS can scavenge H_2_O_2_ to further avoid oxidative damage to the algal cells^66^. Moreover, Zhang et al.^67^ verified the ability of CPS in *Thalassiosira pseudonana* on weakening toxicity of CdSe quantum dots. We also observed a CPS rise in *D. flos-aquae* after WMPAE treatment (Fig. 8). The CPS contents in all treatment groups increased markedly after 24 h. The relative CPS contents of the treatment groups when compared to the control group after 72 h of exposure were 4.98 times (1.8 g L^−1^ WMPAE), 7.82 times (3.6 g L^−1^3 WMPAE), and 11.48 times (5.4 g L^−1^ WMPAE) higher. These large increases in CPS contents are consistent with findings in *Microcystis* cells, in which two morphological *Microcystis* strains showed obvious increases in CPS in response to exposure to *Acorus calamus* root hexane extract^43^. The gradual but pronounced increase in CPS content of *D. flos-aquae* hints that the initial response of algal cells to WMPAE stress is to synthesize more CPS, allowing stronger interactions between its hydrophobic components and the toxic substance^66,68,69^.

## Conclusions

The algacidal efficiency of WMPAE on *D. flos-aquae* blooms were investigated in the present study. The analyses explored several physiological and photosynthetic indexes, as well as the inhibitory mechanisms involved. Our results indicate that WMPAE can inhibit growth, decrease cell viability, induce intracellular oxidative stress, and cause intracellular damage in *D. flos-aquae*. Furthermore, WMPAE also degrade Chl a in algal cells and intensively impair the photosynthesis in *D. flos-aquae*. Carotenoid (in the low exposure level only) and CPS contents in all treatment groups were stimulated. These responses represent attempts to increase the resistance of the algal cells to WMPAE-induced stress. Overall, our results suggest that WMP, an agricultural waste product, is a promising candidate for the control of *D. flos-aquae* blooms, which pose environmental and human health threats. However, for successful administration more detailed research is required in the future e.g., allelochemical analyses.

## Data Availability

The data are available from the corresponding author on reasonable request.
